# Associations Between Fatigue and Sleep Among Night‐Shift Nurses: Mediating Role of Depressive Symptoms and Social Support

**DOI:** 10.1155/jonm/5828649

**Published:** 2026-07-30

**Authors:** Yingying Gu, Qiqi Huang, Shuhong Ni, Caijun Dai, Wenwei Ren

**Affiliations:** ^1^ Department of Psychiatry, The First Affiliated Hospital of Wenzhou Medical University, Wenzhou 325000, Zhejiang, China, wzhospital.cn; ^2^ Pediatric Nursing Unit, The First Affiliated Hospital of Wenzhou Medical University, Wenzhou, China, wzhospital.cn; ^3^ Department of Nursing, Jinhua Municipal Central Hospital, Jinhua, Zhejiang, China, jhzxyy.cn; ^4^ Department of Pulmonary and Critical Care Medicine, Jinhua Municipal Central Hospital, Jinhua, Zhejiang, China, jhzxyy.cn

**Keywords:** depressive symptoms, fatigue, night-shift nurses, sleep, social support

## Abstract

**Background:**

This study examined the association between fatigue and sleep disturbance among night‐shift nurses during the COVID‐19 pandemic and the mediating roles of depressive symptoms and social support.

**Methods:**

A cross‐sectional survey was conducted involving 1667 night‐shift nurses from multiple hospitals. The Pittsburgh Sleep Quality Index (PSQI), Fatigue Scale‐14, Hospital Anxiety and Depression Scale, and Social Support Rating Scale were used for assessment. Correlation analysis and mediation effect analysis were performed using SPSS 26.0 and PROCESS macro.

**Results:**

Poor sleep (PSQI ≥ 6) was reported by 70.7% of nurses. Increased workload (89.7%) and fear of infection (58.1%) were significantly associated with worse sleep (both *p* < 0.05). Fatigue correlated positively with depression (*r* = 0.607, *p* < 0.001) and sleep disturbance (*r* = 0.550, *p* < 0.001) and negatively with social support (*r* = −0.242, *p* < 0.001). Mediation analysis identified three pathways: depressive symptoms alone (30.25% of the total effect), social support alone (0.80%), and the sequential depression ⟶ social support path (2.23%).

**Conclusions:**

Among night‐shift nurses, fatigue is directly and indirectly associated with poorer sleep quality via three mediating pathways: depressive symptoms alone, social support alone, and the sequential effect of depressive symptoms on social support. Interventions should address these pathways to improve sleep in night‐shift nurses under high‐load public health emergency contexts.

**Implications for Nursing Management:**

For night‐shift nurses facing excess workload during public health emergencies, managers could adopt a tiered strategy: (1) optimize shift schedules to reduce fatigue and (2) strengthen peer and family support systems. Regular monitoring of sleep and fatigue indicators is recommended to evaluate intervention effectiveness under such high‐stress contexts.

## 1. Introduction

Although the acute phase of the COVID‐19 pandemic has passed, its detrimental effects on healthcare workers’ well‐being persist [1, 2]. Nurses have reported significantly heightened work‐related fatigue [3, 4] and increased prevalence of depressive and anxiety symptoms during this period [5, 6]. These challenges are further exacerbated for night‐shift nurses, a population already at elevated risk for sleep disorders [7, 8] and persistent fatigue [9] due to circadian misalignment inherent to their work schedules [10]. Pandemic‐related stressors—such as chronic understaffing, increased workloads, and fear of infection—likely intensified the preexisting, vicious cycle between fatigue and poor sleep among this group [11, 12], constituting a pressing occupational health concern.

The relationship between sleep and fatigue is fundamentally bidirectional. While circadian disruption and poor daytime sleep drive fatigue [9, 13, 14], fatigue itself can severely impair sleep initiation and maintenance [15–18]. This cyclical dynamic creates a self‐perpetuating loop that deteriorates both conditions over time. Although this bidirectional link is recognized, scant research has elucidated the specific psychosocial pathways that mediate the impact of fatigue on sleep disturbance—knowledge that is crucial for nursing management to develop targeted interventions. However, the potential sequential mediation role of depressive symptoms and social support—whereby fatigue erodes psychological resources, which in turn diminishes protective social resources, ultimately worsening sleep—remains untested among night‐shift nurses.

With conservation of resources (COR) theory serving as the core theoretical framework of this study, we propose a testable serial mediation model grounded in this classic theory [19]. Within this framework, we conceptualize fatigue as the depletion of a core energy resource (primary core resource in COR), which may trigger subsequent loss in intrapersonal psychological resources manifested as elevated depressive symptoms. This psychological depletion can then impair the acquisition and utilization of external conditional resources represented by social support [20, 21], ultimately worsening sleep quality. This hypothesized hierarchical resource‐loss cascade (“fatigue ⟶ depressive symptoms ⟶ social support ⟶ sleep disturbance”) is derived strictly from the resource depletion law of COR theory. This hypothesized cascade aligns with evidence showing high rates of depression among nurses [22], which can inhibit social engagement and help‐seeking [20, 21]. The COVID‐19 pandemic, with its associated surge in workload and fear of infection (e.g., 89.7% of our sample reported increased workload), represents a context that likely exacerbates this entire resource loss spiral, making its investigation timely and critical.

Therefore, grounded in the theoretical framework of COR theory, this study aims to test this hypothesized chain of resource loss by examining whether depressive symptoms and social support sequentially mediate the relationship between fatigue and sleep among night‐shift nurses. Elucidating this pathway will provide an empirical foundation for designing targeted, multilevel interventions to improve sleep and mental health within this vital workforce.

## 2. Materials and Methods

### 2.1. Study Design and Participants

This cross‐sectional study used an online survey platform (https://www.wjx.cn) with secure data encryption to ensure participant confidentiality [23]. Recruitment occurred through WeChat groups using QR codes, allowing voluntary participation with informed consent. The study was approved by the Ethics Committee of Jinhua Municipal Central Hospital (No. 20222220101). From August to September 2022, we recruited 2140 nurses across 11 hospitals in Jinhua. After excluding 446 day‐shift nurses and 27 nonnursing staff, the final sample included 1667 night‐shift nurses. The eligible participants were as follows: registered nurses ≥ 18 years old; currently working night shifts; and capable of completing the questionnaire independently. Individuals refusing to participate in the survey were excluded from the research. In the present study, we operationalized shift work with a specific time frame, defining night shifts as the period from 23:00 to 08:00. Consistent with our prior publication [24, 25], this study employed the same research design, methods, and data collection approaches. Nevertheless, it differs with respect to the key indicators examined and the core research objectives.

### 2.2. Demographic Characteristics

Demographic and pandemic‐related occupational variables (age, sex, marital status, education, years of nursing experience, professional title, COVID‐19 exposure level, increased workload, and fear of COVID‐19 infection) were collected via a standardized information sheet. We defined nurses’ COVID‐19 exposure level according to their working department via a single questionnaire item. Nurses working in fever clinics, infectious disease departments, departments of respiratory and critical care medicine, emergency departments, or isolation wards were assigned to the high‐exposure group, while those from all other departments were categorized into the nonexposure (low‐exposure) group.

### 2.3. Questionnaires

#### 2.3.1. Fatigue Scale

Fatigue was assessed using the 14‐item Fatigue Scale (FS‐14) [26], which measures physical (items 1–8) and mental fatigue (items 9–14) through dichotomous (yes/no) responses. Total scores range from 0–14, with higher scores indicating greater fatigue severity. The Chinese version demonstrates good reliability (Cronbach’s *α* = 0.863; McDonald’s *ω* = 0.84) [27, 28]. In this sample, the FS‐14 showed good internal consistency, with a Cronbach’s *α* value of 0.837.

#### 2.3.2. Pittsburgh Sleep Quality Index (PSQI)

Sleep quality was assessed using the PSQI [29, 30], a 19‐item instrument evaluating seven sleep components over the past month: subjective quality, latency, duration, efficiency, disturbances, medication use, and daytime dysfunction. Global scores (0–21) were categorized as follows: ≤ 5 (good), 6–10 (mild impairment), 11–15 (poor), and 16–21 (severe impairment). The scale demonstrated strong reliability (*α* = 0.83) and validity (0.83) [29]. In this sample, the PSQI showed good internal consistency (Cronbach’s *α* = 0.856), with its global score used to reflect overall sleep quality (higher scores indicate poorer sleep).

#### 2.3.3. The Hospital Anxiety and Depression Scale (HADS)

The HADS [31] was administered to screen for emotional distress, comprising two 7‐item subscales (depression and anxiety) scored 0–3 per item (total range: 0–21 per subscale). Using the standard > 7 cutoff [32], the instrument demonstrates 82% sensitivity and 72% specificity for depression detection. Scores were categorized as follows: 0–7 (normal), 8–10 (possible depression), and 11–21 (probable depression) [31]. The scale showed excellent reliability (*α* = 0.85; *ω* = 0.82) in validation studies [33]. In the present study sample, the HADS showed good internal consistency, with a Cronbach’s *α* value of 0.908.

#### 2.3.4. Social Support Rating Scale (SSRS)

Social support was assessed using a validated Chinese version of the SSRS [34, 35]. This 10‐item instrument measures three domains: (1) objective support, (2) subjective support, and (3) support utilization, with total scores ranging from 12 to 66. Higher scores reflect greater support levels, categorized as low (< 23), medium (23–44), or high (45–66). The scale demonstrated excellent reliability (full‐scale *α* = 0.81; subscales: 0.878–0.903) and confirmed factor structure [35]. In the present study sample, the SSRS showed good internal consistency, with a Cronbach’s *α* value of 0.801.

### 2.4. Data Analysis

All statistical analyses were performed using SPSS Version 26.0. Continuous variables were expressed as mean ± standard deviation following verification of normal distribution using the Kolmogorov–Smirnov test. Categorical variables were presented as frequencies with corresponding percentages. Chi‐square tests compared categorical demographic and pandemic variables across good (PSQI ≤ 5) and poor sleep (PSQI ≥ 6) groups (Table 1). One‐way ANOVA tested continuous score differences across three‐level occupational and educational subgroups. Pearson correlations examined linear relationships among fatigue, depressive symptoms, sleep quality, and social support (Table 2).

**TABLE 1:** Demographic characteristics and the distribution of sleep (*n* = 1667).

Variables	*n* (%)	PSQI score		*χ* ^2^/*F*	*p*
		≤ 5 (*n* = 489)	≥ 6 (*n* = 1178)		
Gender				3.503	0.061
Female	1613 (96.8)	467 (95.5)	1146 (97.3)		
Male	54 (3.2)	22 (4.5)	32 (2.7)		
Age (year)				4.034	0.133
≤ 29	899 (53.9)	282 (31.4)	617 (68.6)		
30–39	630 (37.8)	168 (26.7)	462 (73.3)		
≥ 40	138 (8.3)	39 (28.3)	99 (71.7)		
Marital status, *n* (%)				1.314	0.518
Married	771 (46.3)	221 (28.7)	550 (71.3)		
Single	868 (52)	262 (30.2)	606 (69.8)		
Other	28 (1.7)	6 (21.4)	22 (78.6)		
Education level, *n* (%)				2.815	0.245
College degree or lower	624 (37.4)	196 (31.4)	428 (68.6)		
Undergraduate degree	1036 (62.1)	290 (28.0)	746 (72.0)		
Master or higher	7 (0.4)	3 (42.9)	4 (57.1)		
Professional title, *n* (%)				2.334	0.311
Primary title	842 (50.5)	259 (30.8)	583 (69.2)		
Intermediate title	692 (41.5)	189 (27.3)	503 (72.7)		
Senior	133 (8)	41 (30.8)	92 (69.2)		
Exposure level, *n* (%)				0.314	0.575
High	485 (29.1)	147 (30.3)	338 (69.7)		
Low	1182 (70.9)	342 (28.9)	840 (71.7)		
Years of work experience (year)				3.808	0.051
< 10	1090 (65.4)	337 (30.9)	753 (69.1)		
≥ 10	577 (34.6)	152 (26.3)	425 (73.7)		
Fear of infection, *n* (%)				7.401	**0.007**
Yes	968 (58.1)	259 (26.8)	709 (73.2)		
No	699 (41.9)	230 (32.9)	469 (67.1)		
Nursing workload				14.848	**0.001**
Reduced	39 (2.3)	14 (35.9)	25 (64.1)		
Normal	134 (8.0)	58 (43.3)	76 (56.7)		
Increased	1494 (89.7)	417 (27.9)	1077 (72.1)		

*Note:* Exposure level was stratified by workplace. High‐exposure settings include fever clinics, infectious disease departments, respiratory and critical care medicine departments, emergency departments, and isolation wards; all other wards were classified as low‐exposure departments. The bolded numbers represent statistically significant *p* < 0.05.

**TABLE 2:** Correlations between observed variables (*n* = 1667).

Variables	1	2	3	4
1. Fatigue	1			
2. Depression	0.607^∗∗∗^	1		
3. Social support	−0.242^∗∗∗^	−0.333^∗∗∗^	1	
4. Sleep	0.550^∗∗∗^	0.519^∗∗∗^	−0.249^∗∗∗^	1

^∗∗∗^ *p* < 0.001.

To test the hypothesized mediation model, we conducted a mediation analysis using PROCESS macro (Model 6) in SPSS with the following specifications: fatigue as the independent variable, sleep as the dependent variable, and both depressive symptoms and social support as sequential mediators. We adjusted for increased workload and fear of infection as covariates (both *p* < 0.05, Table 1). These two pandemic‐specific stressors were significantly linked to poor sleep, allowing us to isolate the independent mediating effects of depressive symptoms and social support. Other demographic and occupational variables showed no significant sleep associations (all *p* > 0.05, Table 1) and were excluded to preserve statistical power. Mediation analyses were conducted using 5000 bootstrap samples to estimate 95% confidence intervals. Effect sizes for mediation pathways were computed as the ratio of indirect effects to total effects, expressed as percentages. Two‐tailed *p* values < 0.05 were considered statistically significant, with mediation effects considered significant when the bootstrap confidence intervals did not include zero.

## 3. Results

### 3.1. Demographic and Descriptive Characteristics

A total of 1667 night‐shift nurses (1613 females and 54 males) were included in this study. As presented in Table 1, majority of participants were ≤ 39 years old (91.8%, *n* = 1529), unmarried (52.0%, *n* = 868), and held a college or undergraduate degree (99.5%, *n* = 1660). Regarding professional titles, 50.5% (*n* = 842) nurses held a primary‐level title, while 41.5% (*n* = 692) held intermediate‐level titles. Most nurses 65.4% (*n* = 1090) had fewer than 10 years of nursing experience. A significant proportion of participants (89.7%, *n* = 1494) reported an increased nursing workload during the COVID‐19 pandemic. Additionally, 58.1% (*n* = 968) expressed concerns regarding the fear of infection. Fear of infection and increased workload were significantly associated with a higher incidence of poor sleep (PSQI ≥ 6) among night‐shift nurses (both *p* < 0.05).

The scores for the core variables among night‐shift nurses were as follows: 7.7 ± 3.50 for the PSQI, 8.34 ± 3.65 for the FS‐14, 35.45 ± 8.04 for the SSRS, and 7.02 ± 4.14 for the HADS‐depression, respectively. Based on PSQI criteria (Table 3), sleep quality distribution was follows: 29.3% (*n* = 488) good (PSQI ≤ 5), 49.9% (*n* = 832) mild impairment (PSQI 6–10), 18.8% (*n* = 313) poor (PSQI 11–15), and 2.0% (*n* = 34) severe impairment (PSQI 16–21). Collectively, 70.7% of participants met the criteria for poor sleep (PSQI ≥ 6).

**TABLE 3:** Participants in perspectives on sleep quality (*n* = 1667).

Items	*n*	%
Subjective sleep quality		
Satisfied	1004	60.2
Unsatisfied	663	39.8
Sleep latency		
0–30 min	659	39.5
31–60 min	599	35.9
> 60 min	409	24.5
Sleep duration		
> 7 h	139	8.3
≤ 7 h	1528	91.7
Habitual sleep efficiency		
85%–100%	991	59.4
75%–84%	570	34.2
0%–74%	106	6.4
Subjective sleep disturbances		
No	149	8.9
Yes	1518	91.1
Use of sleep medication		
Use	213	12.8
Not use	1454	87.2
Daytime functioning		
Yes	1375	82.5
No	292	17.5
Total scores of sleep disturbances		
> 5	1178	70.7
≤ 5	489	29.3

### 3.2. Correlations of all Examined Variables

As summarized in Table 2, all examined variables demonstrated statistically significant associations (*p* < 0.001). Fatigue exhibited strong positive correlations with depressive symptoms (*r* = 0.607) and sleep disturbance (*r* = 0.550), while showing a moderate negative correlation with social support (*r* = −0.242). Depressive symptoms were positively associated with sleep disturbance (*r* = 0.519) and inversely correlated with social support (*r* = −0.333). Social support was negatively associated with sleep disturbance (*r* = −0.249).

### 3.3. Mediation Analysis

**FIGURE 1:**
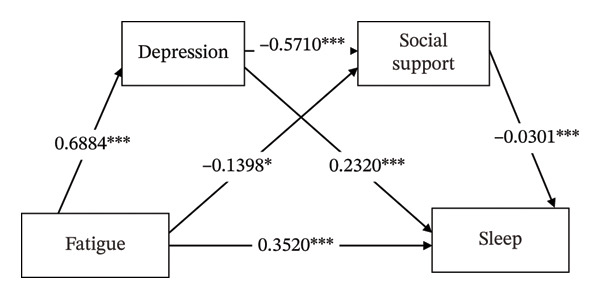
Serial mediation model of fatigue, depressive symptoms, social support, and sleep quality among night‐shift nurses. Note: ^∗∗∗^ *p* < 0.001; ^∗^ *p* < 0.05.

The mediation model, with depression and social support specified as mediators between fatigue and sleep quality, is presented in Figure 1 and Table 4. The sleep outcome variable is the PSQI global score, where higher scores represent poorer sleep quality. The analysis shows a significant total association between fatigue and poor sleep quality (*β* = 0.5276, 95% CI [0.4891, 0.5662]). Effect decomposition demonstrates a substantial direct pathway from fatigue to sleep (*β* = 0.352, 95% CI [0.306, 0.399]), which accounted for 66.72% of the total effect. Additionally, the total indirect effect is significant (effect = 0.1756, 95% CI [0.1428, 0.2081]), explaining 33.28% of the total effect.

**TABLE 4:** Total, direct, total indirect, and specific indirect effects (*n* = 1667).

Structural path	Effect value	Effect size (%)	95% CI	
			Lower	Upper
Total effect	0.5276	100	0.4891	0.5662
Direct effect	0.3520	66.72	0.3055	0.3985
Total indirect effect	0.1756	33.28	0.1428	0.2081
Indirect effect 1	0.1596	30.25	0.1259	0.1925
Indirect effect 2	0.0042	0.80	0.0001	0.0102
Indirect effect 3	0.0118	2.23	0.0041	0.0201

*Note:* Indirect effect 1: fatigue ⟶ depression ⟶ sleep. Indirect effect 2: fatigue ⟶ social support ⟶ sleep. Indirect effect 3: fatigue ⟶ depression ⟶ social support ⟶ sleep.

Further analysis of the specific indirect pathways indicates that the primary mediation occurred through depression (indirect effect 1: fatigue ⟶ depression ⟶ sleep). This pathway yielded an effect value of 0.1596 (95% CI [0.1259, 0.1925]), constituting 30.25% of the total effect. The constituent path coefficients indicate that fatigue was strongly positively associated with elevated depressive symptoms (*β* = 0.6884, *p* < 0.001), and depressive symptoms are significantly positively associated with poorer sleep (*β* = 0.2320, *p* < 0.001).

A secondary significant mediation pathway is identified (indirect effect 3: fatigue ⟶ depression ⟶ social support ⟶ sleep), with an effect value of 0.0118 (95% CI [0.0041, 0.0201]). This pathway accounted for 2.23% of the total effect, illustrating that fatigue is also linked to poorer sleep via a sequential associative pathway: fatigue is positively associated with more severe depressive symptoms (*β* = 0.6884, *p* < 0.001), which in turn are inversely associated with perceived social support (*β* = −0.5710, *p* < 0.001), and lower social support is subsequently associated with worse sleep outcomes (*β* = −0.0301, *p* < 0.001). In contrast, the pathway involving social support alone (indirect effect 2) is also statistically significant (effect = 0.0042, 95% CI [0.0001, 0.0102]), indicating its independent mediating role.

To test the robustness of our findings, we performed a sensitivity analysis by rerunning the serial mediation model with adjustment for increased workload and fear of infection. The results show that all direct and indirect effects remained statistically significant, with consistent effect sizes and 95% CIs excluding zero, confirming the stability of our findings.

## 4. Discussion

To the best of our knowledge, this is the first study to explore the mediating pathways linking fatigue to sleep disturbance in night‐shift nurses during the COVID‐19 pandemic, focusing on the combined roles of depressive symptoms and social support. Our findings reveal three distinct mediating mechanisms underlying this association, which explains the complex interplay between fatigue and sleep problems among night‐shift nurses under public health emergency–related stress.

Consistent with prior work [36–38], we adopted the PSQI to assess sleep quality, while other studies used PHQ‐9, GAD, or the HADS for emotional symptom measurement. Despite differences in assessment tools, the correlational trends of core variables were largely consistent across studies. A number of investigations conducted during the COVID‐19 pandemic have verified the co‐occurrence of fatigue, sleep disturbance, and emotional distress among various populations [36–38]. Similar mediating pathways have also been reported in healthcare worker samples during the pandemic [39–41]. Existing evidence indicates that depressive symptoms function as the primary mediator between fatigue and sleep disturbance, exerting a stronger indirect effect than other psychological factors including anxiety. This is consistent with our finding that depressive symptoms accounted for 30.25% of the total mediating effect. The prevalence of poor sleep quality in our sample reached 70.7%, which is considerably higher than the 43%–48.5% reported in earlier investigations [42, 43]. This discrepancy arises from differences in study samples. While prior studies enrolled nurses working mixed day and night shifts, the current research exclusively recruited nurses on fixed night‐shift schedules.

The COR theory [19] provides a coherent framework for interpreting these findings. We conceptualize fatigue as the initial depletion of a fundamental physical resource (physiological energy), which triggers a resource loss spiral [19]. Quantitatively, the dominant mediating role of depressive symptoms—accounting for 30.25% of the total effect—substantiates this view. Depressive symptoms represent the erosion of a key psychological resource (emotional regulation), which then disrupts sleep through increased rumination and emotional arousal, thereby obstructing energy restoration and perpetuating the spiral [44]. This finding is consistent with prior studies reporting that depressive symptoms mediate the relationship between occupational stress and sleep disturbance [45, 46], further confirming the core bridging role of emotional disorders between occupational stress and sleep problems. The vulnerability of night‐shift nurses is further amplified by circadian misalignment [47, 48], which inherently restricts opportunities for psychological replenishment—a constraint often exacerbated by suboptimal daytime sleep environments, thus deepening the resource deficit.

Furthermore, social support, a core “conditional resource” in COR theory, mediated 0.8% of the effect. A plausible explanation lies in the unique constraints of the COVID‐19 pandemic: infection control measures likely reduced opportunities for peer interaction, while concurrent depressive symptoms may have impaired nurses’ initiative to seek support. This aligns with the principle that a deficit in conditional resources exacerbates core resource loss; fatigue likely diminishes nurses’ capacity to seek or maintain supportive interactions, thereby weakening a vital buffer against sleep disturbance.

A key novel finding is the sequential mediation pathway (fatigue ⟶ depressive symptoms ⟶ social support ⟶ poor sleep), which explains 2.23% of the total effect and provides novel evidence for a hierarchical resource loss process. This pathway elucidates a previously unquantified mechanism: psychological resource depletion (depressive symptoms) actively impairs the acquisition of conditional resources (social support). For instance, a fatigued and depressed nurse may perceive offers of help from colleagues as an emotional burden and thus decline them, a behavior that simultaneously reduces social support and foregoes opportunities for practical resource conservation (e.g., shared workload), thereby accelerating the loss spiral. This finding extends COR theory by empirically demonstrating that psychological resource loss can directly orchestrate conditional resource loss within the demanding context of night‐shift nursing.

### 4.1. Implications for Nursing Management

Our findings reveal that fatigue is associated with poorer sleep among night‐shift nurses via three pathways, with depressive symptoms playing the primary mediating role. We recommend a multitiered management strategy: redesigning rosters for fatigue mitigation, integrating systematic mental health support to address depression, and fostering structured support mechanisms with peers and families. These evidence‐based interventions, coupled with ongoing monitoring, are crucial to safeguard nurse well‐being and sustain clinical performance.

## 5. Conclusion

In summary, this study clarifies the complex pathways through which fatigue correlates with sleep disturbance among night‐shift nurses surveyed during the COVID‐19 pandemic. By identifying depressive symptoms as the primary mediator and verifying the sequential mediating role of social support, our findings provide solid empirical evidence for developing targeted multilevel interventions to protect sleep health for night‐shift nurses experiencing heavy workload amid public health emergencies.

### 5.1. Limitations

This study has the following limitations. First, due to the cross‐sectional design, causal relationships cannot be confirmed. Future longitudinal studies are required to clarify the temporal order and dynamic interactions among fatigue, depressive symptoms, social support, and sleep quality. Second, all data relied on self‐reported questionnaires, which may introduce recall bias. Subsequent research could combine objective tools such as actigraphs to assess sleep status. Third, the sample was recruited from a single region, which limits the generalizability of our findings. Multicenter studies across diverse geographical areas are warranted in the future. Fourth, voluntary online survey recruitment was adopted in this study, which may introduce potential selection bias. Specifically, nurses with poorer sleep quality or more severe fatigue might have been more likely to participate, whereas those with heavier workloads or less available time might have been underrepresented. We attempted to minimize this bias by recruiting participants across 11 hospitals and retaining a large final sample to improve representativeness. In addition, the study was performed during the COVID‐19 pandemic with extra workload and infection concerns, which may inflate variable correlations and mediation effect values; hence, the exact quantitative findings cannot be generalized to night‐shift nurses under conventional working environments, and multicenter longitudinal research across different periods is warranted for further verification.

## Author Contributions

Data analysis and interpretation were performed by Yingying Gu, Caijun Dai, and Wenwei Ren, who also contributed to manuscript drafting. The study design and data collection were overseen by Shuhong Ni and Qiqi Huang. All coauthors participated in critical review of the manuscript.

## Funding

This study was supported by the Wenzhou Science and Technology Bureau Project (Y20220841).

## Disclosure

All authors gave final approval for publication.

## Ethics Statement

This research was conducted in accordance with the ethical principles of the Declaration of Helsinki (1975) and was approved by the Institutional Review Board of Jinhua Municipal Central Hospital (Approval No. 20222220101). All participants provided informed consent before data collection.

## Consent

Please see the Ethics Statement.

## Conflicts of Interest

The authors declare no conflicts of interest.

## Supporting Information

Additional supporting information can be found online in the Supporting Information section.

## Supporting information


**Supporting Information** STROBE Statement—checklist of items that should be included in reports of observational studies.

## Data Availability

The data are available from the corresponding author upon reasonable request.
