# Supplementary material for: Associations Between Fatigue and Sleep Among Night‐Shift Nurses: Mediating Role of Depressive Symptoms and Social Support
Source: J Nurs Manag. 2026 Jul 30;2026:5828649. doi: 10.1155/jonm/5828649 (PMC13424447; doi:10.1155/jonm/5828649)
Supplement: Supplementary file 1 — Supporting Information STROBE Statement—checklist of items that should be included in reports of observational studies. [file JONM-2026-5828649-s001.docx]

STROBE Statement—checklist of items that should be included in reports of observational studies

|  | Item No. | Recommendation | Page  No. | Relevant text from manuscript |
| --- | --- | --- | --- | --- |
| Title and abstract | 1 | (a) Indicate the study’s design with a commonly used term in the title or the abstract | 2 | A cross-sectional survey |
|  |  | (b) Provide in the abstract an informative and balanced summary of what was done and what was found | 2 | Full Abstract |
| Introduction | | | |  |
| Background/rationale | 2 | Explain the scientific background and rationale for the investigation being reported | 3-4 | Introduction section Paragraph 1-3 |
| Objectives | 3 | State specific objectives, including any prespecified hypotheses | 4 | This study aims to test this hypothesized chain of resource loss by examining whether depressive symptoms and social support sequentially mediate the relationship between fatigue and sleep among night-shift nurses. Hypothesized cascade: fatigue → depressive symptoms → social support → sleep disturbance based on Conservation of Resources (COR) Theory. |
| Methods | | | |  |
| Study design | 4 | Present key elements of study design early in the paper | 4 | This cross-sectional study used an online survey platform (www.wjx.cn) with secure data encryption to ensure participant confidentiality |
| Setting | 5 | Describe the setting, locations, and relevant dates, including periods of recruitment, exposure, follow-up, and data collection | 4 | Recruitment time: August to September 2022; Setting: 11 hospitals in Jinhua, Zhejiang Province, China; Cross-sectional one-time data collection with no follow-up period. Night shift defined as 23:00 to 08:00. |
| Participants | 6 | (a) Cohort study—Give the eligibility criteria, and the sources and methods of selection of participants. Describe methods of follow-up  Case-control study—Give the eligibility criteria, and the sources and methods of case ascertainment and control selection. Give the rationale for the choice of cases and controls  Cross-sectional study—Give the eligibility criteria, and the sources and methods of selection of participants | 4 | Eligible participants: registered nurses ≥18 years old, currently working night shifts, capable of completing the questionnaire independently. Exclusions: day-shift nurses, non-nursing staff, participants refusing to join. Participants were recruited via WeChat groups of cooperating hospitals. |
|  |  | (b) Cohort study—For matched studies, give matching criteria and number of exposed and unexposed  Case-control study—For matched studies, give matching criteria and the number of controls per case | N/A |  |
| Variables | 7 | Clearly define all outcomes, exposures, predictors, potential confounders, and effect modifiers. Give diagnostic criteria, if applicable | 4-5 | Independent variable: fatigue (FS-14); Mediators: depressive symptoms (HADS depression subscale), social support (SSRS); Dependent outcome: sleep quality (PSQI, cutoff: PSQI ≥6 = poor sleep). Confounders: increased workload, fear of COVID-19 infection. Cutoff values for all scales were fully stated. |
| Data sources/ measurement | 8* | For each variable of interest, give sources of data and details of methods of assessment (measurement). Describe comparability of assessment methods if there is more than one group | 5 | All data were self-reported via four validated Chinese versions of questionnaires (FS-14, PSQI, HADS, SSRS). Reliability coefficients (Cronbach’s α) of each scale in this sample were reported. All participants received identical online questionnaires, so assessment methods were fully comparable across all subjects. |
| Bias | 9 | Describe any efforts to address potential sources of bias | 6 | Adjusted two major pandemic-related confounders (increased workload, fear of infection) in mediation models |
| Study size | 10 | Explain how the study size was arrived at | 4 | From August to September 2022, we recruited 2140 nurses across 11 hospitals in Jinhua. After excluding 446 day-shift nurses and 27 non-nursing staff, the final sample included 1667 night-shift nurses. |

Continued on next page

| Quantitative variables | 11 | Explain how quantitative variables were handled in the analyses. If applicable, describe which groupings were chosen and why | 5 | Questionnaires section |
| --- | --- | --- | --- | --- |
| Statistical methods | 12 | (a) Describe all statistical methods, including those used to control for confounding | 6 | Data Analysis section |
|  |  | (b) Describe any methods used to examine subgroups and interactions | N/A |  |
|  |  | (c) Explain how missing data were addressed | N/A |  |
|  |  | (d) Cohort study—If applicable, explain how loss to follow-up was addressed  Case-control study—If applicable, explain how matching of cases and controls was addressed  Cross-sectional study—If applicable, describe analytical methods taking account of sampling strategy | 6 | Convenience cluster sampling via hospital WeChat groups was applied; cross-sectional serial mediation model with bootstrap confidence intervals was used to test indirect effects without follow-up data. |
|  |  | (e) Describe any sensitivity analyses | 10 | Results section |
| Results | | | | |
| Participants | 13* | (a) Report numbers of individuals at each stage of study—eg numbers potentially eligible, examined for eligibility, confirmed eligible, included in the study, completing follow-up, and analysed | 7-8 | Table 1 |
|  |  | (b) Give reasons for non-participation at each stage | 4 | Exclusion reasons: holding day-shift position, non-nursing occupation, voluntary refusal to fill out questionnaires. |
|  |  | (c) Consider use of a flow diagram | N/A |  |
| Descriptive data | 14* | (a) Give characteristics of study participants (eg demographic, clinical, social) and information on exposures and potential confounders | 6-7, Table 1 | Reported gender, age, marital status, education, professional title, working years, COVID-19 exposure level, workload change, infection fear; Mean ± SD of FS-14, PSQI, HADS-depression and SSRS total scores; Distribution of sleep quality stratified by demographic and occupational variables. |
|  |  | (b) Indicate number of participants with missing data for each variable of interest | N/A | Missing data for all research variables were not observed or reported in this sample. |
|  |  | (c) Cohort study—Summarise follow-up time (eg, average and total amount) | N/A |  |
| Outcome data | 15* | Cohort study—Report numbers of outcome events or summary measures over time |  |  |
|  |  | Case-control study—Report numbers in each exposure category, or summary measures of exposure |  |  |
|  |  | Cross-sectional study—Report numbers of outcome events or summary measures | 8-9, Table 2 | 70.7% (n=1178) nurses had poor sleep (PSQI ≥ 6); Detailed distribution of each PSQI component (subjective sleep satisfaction, sleep latency, sleep duration, efficiency, disturbance, medication use, daytime dysfunction) with counts and percentages of participants. |
| Main results | 16 | (a) Give unadjusted estimates and, if applicable, confounder-adjusted estimates and their precision (eg, 95% confidence interval). Make clear which confounders were adjusted for and why they were included | 6-11 | Full results section |
|  |  | (b) Report category boundaries when continuous variables were categorized | 5, Table 2 | PSQI cutoff values: ≤5 good, 6–10 mild impairment, 11–15 poor, 16–21 severe impairment; HADS depression cutoff >7 for probable depression; SSRS boundaries: <23 low support, 23–44 medium, 45–66 high support. |
|  |  | (c) If relevant, consider translating estimates of relative risk into absolute risk for a meaningful time period | N/A |  |

Continued on next page

| Other analyses | 17 | Report other analyses done—eg analyses of subgroups and interactions, and sensitivity analyses | 11 | a sensitivity analysis |
| --- | --- | --- | --- | --- |
| Discussion | | | | |
| Key results | 18 | Summarise key results with reference to study objectives | 12 | This study firstly identified three distinct mediating pathways linking fatigue to sleep disturbance among night-shift nurses during COVID-19; Depressive symptoms acted as the primary mediator accounting for 30.25% of total effect, and a novel sequential mediation path (fatigue → depression → social support → poor sleep) was verified, consistent with the COR theory hypothesis proposed in the introduction. |
| Limitations | 19 | Discuss limitations of the study, taking into account sources of potential bias or imprecision. Discuss both direction and magnitude of any potential bias | 14 | Limitations section |
| Interpretation | 20 | Give a cautious overall interpretation of results considering objectives, limitations, multiplicity of analyses, results from similar studies, and other relevant evidence | 12-13 | Results were interpreted under the framework of COR resource loss spiral theory; Compared with previous literature on nurse fatigue and sleep problems to explain higher poor sleep prevalence in this all-night-shift sample; Restricted causal inference due to cross-sectional limitation, only associative relationships could be concluded. |
| Generalisability | 21 | Discuss the generalisability (external validity) of the study results | 13-14 | Findings are applicable to night-shift nurses working in public health emergency overload contexts in similar regional hospitals of China. Multi-center longitudinal research across different geographic areas and non-pandemic periods is required to validate generalizability to broader nursing populations. |
| Other information | |  | | |
| Funding | 22 | Give the source of funding and the role of the funders for the present study and, if applicable, for the original study on which the present article is based | 15 | This study was supported by the Wenzhou Science and Technology Bureau Project (Y20220841). |

*Give information separately for cases and controls in case-control studies and, if applicable, for exposed and unexposed groups in cohort and cross-sectional studies.

**Note:** An Explanation and Elaboration article discusses each checklist item and gives methodological background and published examples of transparent reporting. The STROBE checklist is best used in conjunction with this article (freely available on the Web sites of PLoS Medicine at http://www.plosmedicine.org/, Annals of Internal Medicine at http://www.annals.org/, and Epidemiology at http://www.epidem.com/). Information on the STROBE Initiative is available at www.strobe-statement.org.
